# WIPO Re:Search—A Platform for Product-Centered Cross-Sector Partnerships for the Elimination of Schistosomiasis

**DOI:** 10.3390/tropicalmed4010011

**Published:** 2019-01-09

**Authors:** Callie J. Weber, Joseph Hargan-Calvopiña, Katy M. Graef, Cathyryne K. Manner, Jennifer Dent

**Affiliations:** BIO Ventures for Global Health, 2101 Fourth Avenue, Suite 1950, Seattle, WA 98121, USA; cweber@bvgh.org (C.J.W.); jhargan@bvgh.org (J.H.-C.); kgraef@bvgh.org (K.M.G.); cmanner@bvgh.org (C.K.M.)

**Keywords:** schistosomiasis, neglected tropical diseases, WIPO Re:Search, BIO Ventures for Global Health, cross-sector collaboration, capacity-building, drug discovery, public-private partnerships

## Abstract

Schistosomiasis is an acute and chronic disease that affects over 200 million people worldwide, and with over 700 million people estimated to be at risk of contracting this disease, it is a pressing issue in global health. However, research and development (R&D) to develop new approaches to preventing, diagnosing, and treating schistosomiasis has been relatively limited. Praziquantel, a drug developed in the 1970s, is the only agent used in schistosomiasis mass drug administration (MDA) campaigns, indicating a critical need for a diversified therapeutic pipeline. Further, gaps in the vaccine and diagnostic pipelines demonstrate a need for early-stage innovation in all areas of schistosomiasis product R&D. As a platform for public-private partnerships (PPPs), the WIPO Re:Search consortium engages the private sector in early-stage R&D for neglected diseases by forging mutually beneficial collaborations and facilitating the sharing of intellectual property (IP) assets between the for-profit and academic/non-profit sectors. The Consortium connects people, resources, and ideas to fill gaps in neglected disease product development pipelines by leveraging the strengths of these two sectors. Using WIPO Re:Search as an example, this article highlights the opportunities for the PPP model to play a key role in the elimination of schistosomiasis.

## 1. The Multifaceted Impact of Schistosomiasis

Schistosomiasis is an acute and chronic disease that affects an estimated 219 million people worldwide [1] and results from infection by parasitic trematode worms of the genus *Schistosoma*. Initial infection occurs when humans come into contact with parasite-infested water sources, and the free-swimming larvae of the parasite, the cercariae, penetrate the skin and migrate into the blood in order to mature. Once inside the circulatory system, these juvenile worms mature into the adult stage [2]. In a study conducted by Warren K.S et al. on Yemeni agricultural workers, it was estimated that in the case of *S. mansoni*, adult worms can have a mean lifespan of five to ten years [3,4] and in some cases have been shown to live as long as 40 years [3,5]. While inside the host, the adult worms are capable of reproducing and laying eggs throughout their lifetimes, leading to a state of chronic infection for the host that, depending on the species, can result in symptoms such as abdominal pain, diarrhea, hepatosplenic inflammation, liver fibrosis, and rectal bleeding (*S. mansoni*, *S. japonicum*, and *S. mekongi*), or obstructive disease in the urinary system, haematuria, chronic fibrosis of the urinary tract, and potentially renal dysfunction (*S. haematobium*). In addition to these species-specific symptoms, all species of *Schistosoma* have been associated with co-morbidities such as anemia [4,6]. 

Schistosomiasis is endemic in 78 countries, 42 of which are in the World Health Organization (WHO) African Region [7]. It is estimated that over 700 million people are at risk of contracting schistosomiasis worldwide and over 200 million people are currently infected, making this a pressing issue in global health [8]. There are six species of *Schistosoma* that are largely responsible for human infection. *S. japonicum* occurs only in Asia and *S. mansoni* occurs in sub-Saharan Africa, the Middle East, South America, and the Caribbean; these two species are responsible for intestinal schistosomiasis. *S. mekongi* is primarily restricted to Laos and Cambodia [9], whereas *S. haematobium* occurs predominantly in Africa and the Middle East [4] and leads to various urogenital clinical presentations that include urinary tract fibrosis, obstructive renal failure, and squamous cell carcinoma (SCC) of the bladder [10,11]. The less common *S. intercalatum* [12] and *S. guineensis* live in the rectal veins and are also known to cause disease [13].

Due to the disabling systemic morbidities associated with schistosomiasis, such as anemia, malnutrition, and impaired childhood development [14], it is estimated that 18.3 age-standardized disability adjusted life years (DALYs) per 100,000 population were lost to schistosomiasis for both males and females in 2017 [15]. This not only places an enormous burden on healthcare systems in endemic countries, but also interferes with economic productivity due to the reduced ability of the affected population to perform physical activities and participate in the workforce [14,16,17]. In addition to the considerable impact schistosomiasis has on adult and working populations, it is also important to consider its long-term impacts on the next generation. Schistosomiasis has been associated with reduced functional scores and malnutrition [18]. This can severely affect a child’s ability [19,20,21] to become educated [22], lead a productive life [21,22], and break out of the vicious cycle of disease-related poverty.

A growing threat to people living in *S. haematobium*-endemic countries is schistosomiasis-associated SCC of the bladder [23]. This disease is thought to be caused by the inflammatory reaction triggered by schistosomal eggs deposited in the bladder [24]. Eggs are typically excreted through urination but can remain lodged in the patient’s bladder. The continued exposure to antigen-releasing eggs during the long lifespan of the adult schistosome [25], and the resulting chronic inflammation and augmented cellular proliferation [4,11,26], increase the likelihood of SCC of the urinary bladder [27], which is estimated to occur at a rate of three to four cases per 100,000 [28]. *S. haematobium* is classified as a carcinogenic agent to humans by the International Agency for Research on Cancer (IARC) [29], and thus it is of high priority to develop more efficacious drugs and better diagnostics to stop acute schistosomiasis from leading to other severe and non-reversible bladder pathologies such as cancer. When treated with praziquantel (PZQ), which is the only approved drug for schistosomiasis, pathological lesions present in the urinary tract are eliminated, indicating that the high cost of caring for patients with schistosomiasis-induced bladder cancer can be avoided by addressing schistosomiasis at an early stage [30]. For the purpose of their studies, Botelho et al. estimated that the current annual treatment cost of PZQ per schistosomiasis patient is $0.16 USD, or approximately $17.92 million USD globally [31]. By not addressing schistosomiasis infection at an early stage, it is estimated that *S. haematobium*-associated bladder cancer results in additional treatment costs of at least $20 million USD per year worldwide [31]. These numbers demonstrate the economic benefits that can result from the development of more efficacious drugs that target this disease.

*S. haematobium* infection has also been shown to be disproportionately detrimental to women’s reproductive and sexual health [32]. Infection-associated genital tract damage can lead to infertility [33], stress incontinence, ectopic pregnancy, increased risk of abortion [4], adverse birth outcomes such as low birth weight, and increased infant and maternal mortality [34]. Further, in multiple population-based studies, *S. haematobium* infection has been linked to an increase in HIV infection in women [35,36], with evidence showing that CD4-positive cells in peripheral blood express increased concentrations of HIV co-receptors [37]. This data implies that cells from patients infected by schistosomiasis may be more susceptible to HIV-1 infection.

Although *S. haematobium* has been known to primarily affect the urinary tract, studies have also shown that in males, *S. haematobium* eggs are found in the seminal vesicles and prostate [38]. In a community-based study of genital schistosomiasis in men from Madagascar, *S. haematobium* eggs were detected in 43% of the semen samples, providing preliminary evidence that male genital organs can also be affected by schistosomiasis [39]. The authors of the study hypothesized that egg deposition in a male’s genital organs could potentially lead to an increased accumulation of lymphocytes, eosinophils, and other white cells, which in turn could increase the viral load in semen from males infected with HIV. Along these lines, other population-based studies demonstrated that schistosomiasis has been linked to an increase in HIV infection due to increased concentrations of CD4-positive cells in semen of men with schistosomiasis compared to men who had been cleared of the infection [40]. By successfully detecting and treating schistosomiasis, the global health community is not only removing the burden of this debilitating disease from the population, but also reducing the chances of people suffering from other high-mortality co-morbidities such as HIV.

According to the 5th Progress Report on the London Declaration on Neglected Tropical Diseases (NTDs) [41], the WHO has set a goal of 75% of at-risk populations covered by schistosomiasis control efforts. Regrettably, there has been very little measurable progress made towards elimination [41]. Although strong evidence suggests that PZQ targets schistosome voltage-gated calcium channels, leading to calcium-dependent contraction and paralysis in treated worms [42,43], and the drug has proven to be very useful in controlling morbidity, it is not effective against immature schistosomes [44,45]. Further, it is currently the only drug that is being used in schistosomiasis mass drug administration (MDA) campaigns [46], thus increasing the risk that schistosomes will develop resistance. While it is imperative that new drugs be developed to continue treating infected and at-risk populations, there has not been significant progress in developing alternative treatments since the development of PZQ by Merck KGaA in the 1970s. In fact, due to limited commercial incentives, very little has been done by the for-profit sector in terms of developing new diagnostics, drugs, and vaccines and fueling the early-stage product development pipeline for schistosomiasis. Although these hurdles, among others, currently stand in the way of the elimination of schistosomiasis, the global community is determined to identify solutions through cross-sector partnerships that not only distribute the risk of product development over various stakeholders, but also engage worldwide expertise. While the barriers are high, and the challenge is significant, this article focuses on the incredible efforts being employed in product research and development (R&D) through cross-sector collaborations to make progress toward elimination of schistosomiasis, and the critical roles public-private partnerships (PPPs) play in making that a reality.

## 2. Limitations of Current Control Efforts and Product Gap Analysis

Current control efforts for schistosomiasis include education programs to promote preventive behavioral and lifestyle changes, such as limiting contact with infected waters, boiling fresh water prior to drinking or bathing, wearing DEET (diethyltoluamide) insect repellent, and implementing vigorous towel drying to prevent parasites from penetrating the skin [47]. However, such preventive strategies focus on decreasing the individual risk of infection, rather than prevention on a large scale. Another approach is to control the host snail population through the administration of effective molluscicides, habitat modification, and biologic control strategies such as introducing competing snail species or natural predators [48]. Despite resulting in high snail mortality, the administration of molluscicides must be replicated twice annually to be effective, and thus is both expensive and (along with habitat modification and biologic control strategies) potentially detrimental to the environment [48]. Concurrent with educational and host snail-targeted interventions, the expansion of the drug, diagnostic, and vaccine development pipelines are crucial to systematically prevent and combat schistosomiasis. These development efforts are the primary focus of this article. 

Schistosomiasis is endemic in 78 countries, and mass-scale administration of PZQ is the primary method of both treatment and prevention in all of the affected nations. Since 2012, MDA, or preventive chemotherapy, has been mandated by the governments of 52 endemic countries, covering approximately 250 million people [47]. 

The MDA movement is rooted in the 2001 World Health Assembly (WHA) resolution calling for the global scale-up of preventive chemotherapy to at-risk population groups for the control of schistosomiasis morbidity [49]. At-risk population groups include school-aged children in endemic areas, adults with occupational risk due to contact with infested waters, and, in many instances, entire communities living in areas with high disease prevalence [47]. In the absence of a preventive vaccine, the annual administration of PZQ is recognized as an accepted schistosomiasis prevention strategy for both children and adults, even during pregnancy and lactation [50]. Further, Merck KGaA and others are reformulating PZQ to develop a pediatric PZQ to expand MDA coverage to include children as young as three to six months old [51]. Efforts to expand the reach of MDA programs to rural communities have received international support, with the objective of reducing schistosomiasis morbidity in school children and funding national control programs that have been widely adopted. For example, in 2012, a five-year grant from the END Fund and Children’s Investment Fund Foundation (CIFF) supported the Kenyan government’s implementation of a nationwide school-based deworming program that included annual administration of PZQ to all school children in schistosomiasis-endemic areas [52,53]. After just two years, the prevalence of *S. haematobium* fell from 18% to 7.6%, according to a 2014 survey that randomly tested students from 60 schools selected across 16 counties in the Nyanza, Rift Valley, Western, and Coast regions of Kenya [53]. All PZQ distributed through this program was donated by Merck KGaA, which recently committed to donate 250 million tablets of PZQ per year indefinitely to further support global MDA efforts [54,55]. 

Efforts put forth for MDA programs have not gone unrewarded. In combination with snail-population control efforts, MDA programs have resulted in the WHO declaring that schistosomiasis is no longer a significant public health issue in several countries including Iran, Japan, Jordan, Mauritius, Morocco, Puerto Rico, and Tunisia [47,48]. 

Despite such successes, MDA programs face significant challenges due to inadequate infrastructure and drug coverage in rural at-risk communities, poor drug compliance, and insufficient monitoring and evaluation [47,56]. A recent WHO publication capturing data from 38 endemic countries reported that 70.9 million school-age children and 18.3 million adults were treated with PZQ for schistosomiasis in 2016, although this only covers 53.7% and 14.3% of at-risk school-age children and adults respectively. The coverage was calculated by dividing the number of children requiring PZQ and treated, by the total number of children in need of PZQ (“WHO 2017 Report on 2016 Treatment of Schistosomiasis”) [57]. 

Even with an unlimited supply, optimal distribution, and high patient compliance in MDA campaigns, PZQ itself has limitations. PZQ was selected as the best treatment option for MDA due to its mild side effects, affordability, single-dose administration, and proven efficacy against every human schistosome species [42,43,58,59]. However, it is important to note that to optimize efficacy, PZQ is recommended to be administered in three 20 mg/kg doses in a single day, but to promote single-dose administration, MDA campaigns supply a single 40 mg/kg daily dose, which contributes to lower treatment efficacy [58]. According to a meta-analysis of 55 published studies, the 40 mg/kg daily dose is justified as a reasonable compromise, although the cure rate varies significantly between species, ranging from 94.7% for *S. japonicum* and 63.5% for mixed *S. haematobium/S. mansoni* infections [60]. Multiple field studies over the last two decades have reported cure rates as low as 52% when administering the recommended 40 mg/kg single dose [56,61,62]. Mild side effects, which include dizziness, nausea, headaches, and diarrhea, further hinder patient compliance [47,56]. In a 2014 tolerability study involving 12,435 participants, incidence of adverse events ranged from 2.3% for urticaria to 31.1% for abdominal pain [60]. This is particularly important to note as children carry most of the schistosomiasis burden and PZQ MDA campaigns often target school-aged children [63]. Additionally, PZQ is not effective against juvenile schistosomes. To ensure parasite clearance and to combat reinfection, repeated treatments are common in endemic areas, although lower efficacy of PZQ is observed in subsequent treatments because surviving juvenile schistosomes experience reduced susceptibility to PZQ as adults [42,62]. 

Despite its drawbacks, PZQ remains the best available option for both prevention and treatment of schistosomiasis, and thus has global support to improve its access and distribution to all endemic populations. However, this complete reliance on a single drug has raised global concerns about the development of PZQ drug resistance [56,64]. The severity of this issue is compounded by the longevity of MDA programs, which may promote the persistence of drug resistance because reductions in PZQ susceptibility are heritable [43,65]. Adult worms with reduced PZQ susceptibility have been found to have higher basal levels of ABC multidrug transporters, suggesting that they remove PZQ along with metabolic toxins by translocating substrates across the cell membrane [43,62,65]. To combat this, studies suggest that co-administration of ABC multidrug transporter inhibitors with PZQ may restore the efficacy of PZQ against adult *S. mansoni* [43,62,65]. To continue to diversify treatment options beyond monotherapy PZQ, the co-administration of PZQ with artemisinin-based derivatives, such as artemether, artesunate, and dihydroartemisinin, are also being explored and have demonstrated higher efficacy than treatment with PZQ alone [66]. For example, a preclinical study in China found that the combined treatment of PZQ and artemether reduced total *S. japonicum* worm burdens by 79–92% compared to a total worm burden reduction of 28–66% when treated with PZQ alone. The study was conducted in rabbits infected with 7- to 12-day-old schistosomula and 42-day-old adult schistosomes [66]. Because PZQ and artemether target different developmental stages of the parasite (PZQ is only effective against adult-stage *Schistosoma* and artemether acts against juvenile worms), their combined administration targets both developmental stages and demonstrates higher efficacy than monotherapies with either drug [42,66]. However, the high inherent fail rate of PZQ (previously cited at 40%) makes it extremely difficult to accurately measure the efficacy of co-administered treatment regimens or the global prevalence of drug resistance.

Despite more than 30 years of scientific and technological advances, the management of schistosomiasis remains unchanged since the development of PZQ in the early 1970s [58]. There is a need to improve current approaches by developing an integrated and intersectional method to sustainably prevent, diagnose, and treat schistosomiasis [56,61]. An antischistosomal vaccine would halt the overuse of PZQ as both a method of prevention and treatment, limit the geographic spread of schistosomiasis, and reduce the high rates of reinfection. Concurrently, the drug development pipeline must be expanded to include a diversified set of compounds that perturb novel molecular targets of PZQ-resistant parasites and that are active against both adult and immature schistosomes. To permit detection of lower-level infections as disease burden and intensity decrease due to successful interventions, highly sensitive, point-of-care (POC) diagnostics are needed. Accurate diagnostics are required for on-site detection of infection before administering treatment, as well as for certification of parasite clearance to avoid unnecessary and prolonged treatment regimens. This two-pronged diagnostic approach will maximize the efficiency of drug administration and distribution.

## 3. Product Development Pipelines

### 3.1. Vaccine Development

Despite increased education and MDA efforts, parasite transmission models predict that, based on the current levels of disease prevalence, the WHO’s goal to control schistosomiasis morbidity by 2020 will not be attainable through MDA efforts alone [67]. Incidence rates could be significantly reduced by implementing strategies to prevent initial infection and reinfection. Thus, there is a need for a more permanent method of prevention than mass administration of PZQ. In recognition of this high-need, high-impact development gap, there have been increased efforts toward the development of an antischistosomal vaccine over the past two decades. In a 2013 meeting to develop Preferred Product Characteristics (PPC) for a schistosomiasis vaccine held by the National Institute of Allergy and Infectious Diseases (NIAID), it was agreed that a vaccine should reduce the overall worm burden by at least 75% and reduce egg excretion by close to 75% in order to be considered effective [68]. Currently, there are 10 vaccines in the development pipeline: Five in preclinical; three in Phase I; one in Phase II; and one in Phase III (Table 1) [69,70,71]. 

The French National Institute of Health and Medical Research (INSERM) and Institut Pasteur took Bilhvax^®^ (Sh-GST28; Phase III), a recombinant *S. haematobium* vaccine, through Phase I clinical testing at Lille University Hospital in France and Phase II in Saint-Louis, Senegal [72,73]. Following positive results, Bilhvax^®^ has progressed into Phase III clinical studies, sponsored by INSERM, in the Saint-Louis Region of Senegal as a potential pediatric vaccine candidate against urinary schistosomiasis, with publication of the trial results pending [74]. 

Sm-14 (Phase II) is being developed in partnership between the Oswaldo Cruz Foundation (FIOCRUZ), the Brazilian governmental financial agency (FINEP), and Alvos Biotecnologia as a vaccine candidate against both *S. mansoni* and *S. haematobium* [72,75]. The Phase I clinical trial was completed in 2014 in Rio de Janeiro, Brazil, and the candidate advanced into Phase II clinical trials in the Saint-Louis Region of Senegal in collaboration with the Infectious Disease Research Institute (IDRI). The Phase II study was completed in June 2017, with publication pending [75,76]. 

Alhydrogel^®^ (Sm-TSP-2; Phase I), and Sm-97 paramyosin (Phase I), are both recombinant *S. mansoni* vaccines. Alhydrogel^®^ was developed in partnership between the Sabin Vaccine Institute and Texas Children’s Hospital Center for Vaccine Development to target intestinal schistosomiasis caused by *S. mansoni* [77,78]. After 18 years of combined efforts, the vaccine is now entering Phase Ib development with continued efforts to be led by the Baylor College of Medicine and Texas Children’s Hospital Center for Vaccine Development [77,78]. The ongoing Phase Ib dose-escalating clinical trial, sponsored by the NIAID, is currently in recruitment phase and will be conducted in the *S. mansoni*-endemic area of Americaninhas, Brazil [79]. 

Calpain^®^ (Sm-p80; Phase I) has been rapidly progressing through the development pipeline due to the targeted efforts of a team of researchers at Texas Tech University, and is poised to enter Phase I clinical trials in 2019 [80]. Sm-p80 is a highly immunodominant antigen in the surface membranes of the worms, and the vaccine works by disrupting the schistosome’s mechanisms to evade the host’s natural immune response [81]. A recent study demonstrated that Calpain^®^ significantly reduced both the quantity and size of the *S. mansoni* hepatic egg load in vaccinated baboons, and investigators are moving forward to conduct a large-scale double-blinded experiment to validate the vaccine’s transmission-blocking potential in baboons [80,81]. 

Although the vaccine development pipeline is expanding, the established pathway to market is both expensive and time-consuming. At the moment, the majority of pipeline vaccines only target a single species of schistosome (primarily *S. mansoni*). Ideally, the development pipeline would feature a vaccine effective against the infective form of the parasite for all schistosome species found in humans. However, until such a product has been discovered, there is a continued need to expand and diversify the vaccine pipeline to include products targeting immunogenic antigens that are conserved across all species. 

### 3.2. Diagnostic Development 

Innovative, effective, and precise diagnostics are particularly important during schistosomiasis elimination, when it is necessary to identify geographic areas where transmission has been interrupted and ensure that the disease is no longer present in the population. Paralleling the vaccine development pipeline, efforts have also been made to improve the accurate diagnosis of schistosomiasis at low levels of infection to best direct and inform key decisions throughout elimination programs: (1) Mapping prevalence—establish a baseline disease prevalence to inform MDA strategy; (2) monitoring the impact of MDA—track disease prevalence; (3) inform reduction/stopping of MDA—determine when morbidity reduction targets have been achieved; (4) post-elimination surveillance—detect the re-emergence of the disease (PATH, Diagnostics for Neglected Tropical Diseases). A single universal diagnostic is unable to inform the entire elimination process, thus there is a need to develop diagnostic tools of varying levels of field implementation and sensitivity to be effectively partnered and applied with each step of schistosome elimination programs (Table 2) [82] (PATH, Diagnostics for Neglected Tropical Diseases).

The below analysis focuses on clinically available diagnostic tests. Currently, the most commonly practiced method of diagnosis for all schistosome species is microscopic evaluation and detection of parasite eggs in human urine or stool, for which WHO recommends a polycarbonate filtration technique or the Kato-Katz (KK) fecal smear, respectively [83,84,85]. However, the KK technique is not sufficiently sensitive for low-grade infections and has been repeatedly shown to underestimate disease burden in areas where transmission has declined, and egg burdens are low [85]. Further, egg shedding is often not observed during an initial infection or in cases of low-level infection, due to the PZQ-induced sterilization of worm pairs or the survival of single-sex worm populations following PZQ treatment [83]. Following multiple rounds of PZQ MDA, low levels of infection will become more common, and thus new diagnostic tools with higher specificity and sensitivity are needed to ensure the accurate detection of schistosomiasis. For instance, following a very successful MDA program in Morocco, there have been zero reported cases of urogenital schistosomiasis from *S. haematobium* since 2004. However, when testing the efficacy of three newly developed diagnostic methods—two commercially available antibody tests (haemagglutination and enzyme-linked immunosorbent assay [ELISA] formats) and a lateral flow antigen strip—it was found that some citizens remained infected with *S. haematobium* worms that were simply not producing eggs [83]. There is a need to develop and validate monitoring diagnostics to accurately detect the re-emergence of schistosomiasis in areas where previous MDA campaigns have been successful. Moreover, there remains a need for the development of highly sensitive diagnostics to complement MDA programs that often result in lower levels of eggs in order to inform the decisions to stop MDA. 

To identify the best technology to fill the need for accurate disease monitoring, recent studies have conducted comparative analyses of available molecular and immunological diagnostic methods that do not rely on egg counting [83,85,86]. ELISAs are able to accurately monitor low-grade infection through the detection of antibodies or antigens and can be adapted for large-scale implementation. Antibody-based technology is limited due to its inability to differentiate between ongoing and previous infections, as the detected antibodies remain present even after the clearance of parasites [86]. However, this may be useful for post-elimination surveillance by monitoring antibody prevalence in age cohorts that were born after elimination was certified; if antibodies are detected in this youth population, then there has been a re-emergence of the disease [82]. An alternative approach is the development of molecular diagnostics to detect *Schistosoma* DNA. Although PCR-based diagnostics have been developed with the ability to detect the four main human *Schistosoma* species with high sensitivity and specificity, PCR technology remains too expensive for mass distribution, and implementation is limited to laboratory settings [86,87]. Although ELISA and PCR technologies have promising features, POC immunodiagnostics have been adopted as the most common alternatives to the KK fecal smear. One of the most promising diagnostics is the POC-CCA developed by Rapid Medical Diagnostics [84]. POC-CCA is a commercially available serological assay that is able to detect *Schistosoma* circulating cathodic antigen (CCA), which is produced by adult schistosomes, in either human blood or urine samples [86]. As CCA is a genus-specific glycan, this method is able to detect active infections caused by the four main species of schistosome that infect humans—excluding urogenital schistosomiasis—and has consistently been identified as a more sensitive substitute for KK when estimating a region’s schistosomiasis prevalence [83,84,85,86,88]. However, the POC-CCA diagnostic has high rates of false negatives due to frequent trace CCA results on the test strip. In a 2017 comparative study in Brazil, 461 participants donated feces, blood, and urine samples to comparatively map schistosomiasis prevalence using three diagnostic mechanisms, including the KK fecal smear and POC-CCA diagnostic [83]. The POC-CCA method produced the highest proportion of false-negative results and the lowest proportion of true-positive results, thus demonstrating the need for expanding the product development pipeline and continuing sensitivity testing and validation of available immunodiagnostics [83,84,85]. 

Through improved access to sequencing technology, the *S. mansoni* genome has been successfully assembled and new secretion proteins from both adult worms and eggs have been identified as new potential diagnostic targets [86]. This presents opportunities for continued development and improvement of multiplex serological assays to simultaneously detect *S. haematobium, S. mansoni*, and *S. japonicum*. Continued development of accurate diagnostics will also improve the detection of reduced drug efficacy and the subsequent monitoring of the development of PZQ resistance. To date, the development of new diagnostics for schistosomiasis has been primarily driven by the academic sector, and there is a need to also engage and incentivize the private sector to drive pipeline expansion and product development.

### 3.3. Drug Development 

Due to the widespread dependence on PZQ to combat schistosomiasis, the therapeutic development pipeline is critically limited (Table 3). The only candidate in clinical trials is Co-Arinate FDC^®^, an artemisinin-based combination therapy (ACT) consisting of artesunate and sulfamethoxypyrazine/pyrimethamin that was developed by Dafra Pharmaceuticals as an antimalarial drug [89]. Due to the high rates of co-infection of malaria and *S. haematobium*, artemisinins have been observed to have unexpected antischistosomal effects, particularly against juvenile parasites [89]. Thus, Co-Arinate FDC^®^ was tested in a Phase III clinical trial to compare its efficacy to that of PZQ against *S. haematobium* in children [90]. Although it has a strong safety profile, Co-Arinate FDC^®^ has only partial efficacy against *S. haematobium*, with a 43.9% cure rate, which was lower than the 53% cure rate observed in patients receiving PZQ [89]. These findings indicate the need for the further exploration of the efficacy of various ACT formulations against *Schistosoma* species. Alternative preclinical and discovery stage therapeutic programs include the repurposing of miltefosine (MFS), which is currently a widely adopted treatment for leishmaniasis, and evaluating the efficacy of thioredoxin glutathione reductase (TGR) inhibitors through high-throughput screening [91,92]. MFS was reported to have significant activity against various stages of *S. mansoni* in in vitro studies, thus spurring further exploration of repurposing that drug for the treatment of schistosomiasis [91]. A 2015 preclinical study reported that delivering MFS in lipid nanocapsules allowed for single-dose oral delivery in mice [91].

To date, the majority of drug development efforts have focused on developing derivatives of PZQ, including the development of a PZQ pediatric formulation and deuterated PZQ analogs [93]. However, to prepare for the risk of widespread drug resistance, there is a critical need for a diversified therapeutic pipeline with novel drug targets. 

### 3.4. Call to Action: Expanding Product Development Pipelines 

To effectively combat schistosomiasis on a population-wide basis, intersectional technological innovations—new drugs with novel molecular targets and activity against immature schistosomes; POC diagnostics to detect low-level infections, assess treatment efficacy, and inform treatment decisions; and effective vaccines to prevent initial infection and reinfection—must be developed and made broadly accessible. 

With over 700 million people at risk for infection, there is a clear and pressing need to develop new tools that make schistosomiasis elimination a reality. However, since schistosomiasis largely affects the world’s poorest populations, consumers are unable to pay high prices for new products. This has resulted in limited commercial incentives to develop innovative vaccines, diagnostics, and drugs for schistosomiasis and other diseases of poverty. To develop new products, companies must make substantial monetary investments during the discovery, preclinical, and clinical stages of development [94], and must bear the risk that the resulting product may not receive U.S. Food and Drug Administration (FDA) approval and may fail to make it to market. In order for a company to remain profitable, its products must generate a margin of profit that returns the initial investments made during R&D. In comparison to “high-incentive” diseases with profitable markets such as cancer, hypercholesterolemia, and other cardiovascular diseases, the profitability of the schistosomiasis market and the socioeconomic status of its customers do not align particularly well with the business models of most pharmaceutical and biotechnology companies, particularly if the companies are expected to shoulder the financial risk and resource-heavy investment associated with de novo product development. Although schistosomiasis is a high-priority disease to the WHO due to the number of people who are currently infected and are at risk for infection, the commercial incentives to invest resources into product development for this disease are low and not compatible with many business models. 

Along these lines, a study that profiled the drug and vaccine landscape for neglected diseases between 2000 and 2011 found that out of 850 new registered products, only 4% were indicated for neglected diseases [95], illustrating the massive gaps in product development for neglected diseases. In order to incentivize the private sector and distribute the financial risk associated with bringing a healthcare product to the market for high-priority, low-incentive indications, new product development models have emerged. One such model is the PPP, in which the strengths of the public and private sectors are combined and leveraged to develop breakthrough products while simultaneously redistributing the financial risk involved with product development [96]. An example of a PPP focused on schistosomiasis is the Pediatric Praziquantel Consortium, which is working to develop a pediatric formulation of PZQ for preschool-aged children. The Pediatric Praziquantel Consortium was launched in 2012 by Astellas Pharma, Merck KGaA, the Swiss Tropical and Public Health Institute (TPH), and Lygature. Armed with funding from the Bill & Melinda Gates Foundation and the Global Health Innovative Technology Fund (GHIT), the Consortium identified promising candidates and is currently optimizing its formulation before moving into clinical trials [97]. Having the capabilities for a Phase I-III clinical development program that includes FDA recommendations for pediatric development, the Consortium aims to have its pediatric-specific product available by 2020 [97]. With approximately 25 million preschool-aged children requiring treatment for schistosomiasis [97], the need for a pediatric formulation of PZQ is pressing, but not highly profitable. Through the Pediatric Praziquantel Consortium, the private and public sectors are working together to fill this high-priority product development gap by contributing specific know-how. Merck KGaA is the sponsor of the clinical trials and will be providing support and resources in the areas of preclinical, clinical, manufacturing, regulatory, and access, while Astellas provides advice on clinical development and pharmacokinetic modeling. Scientists from Swiss TPH, as well as other academic institutions, will contribute extensive experience in helminth biological and pharmacological research; epidemiology; and clinical research in endemic regions. Further, by pivoting from the current donation-based PZQ-MDA model towards a more sustainable pricing model, the Pediatric Praziquantel Consortium plans to make the product available on a not-for-profit basis. The Pediatric Praziquantel Consortium is exploring alternative business models and will work with relevant stakeholders to examine ways of providing access to its novel formulation in an affordable manner. This type of partnership demonstrates that, through the sharing of resources and expertise between the private and public sectors, it is possible to develop products for a market that historically has not been highly profitable.

Another type of collaborative model that fits under the umbrella of a PPP is the not-for-profit product development partnership (PDP). PDPs focus on product development and uncoupling the cost of R&D from the price of medicines, thereby resulting in a more affordable product [98]. Many PDPs are also PPPs, as they work with the pharmaceutical industry, academic research institutions, and other not-for-profit organizations. PDPs such as the Drugs for Neglected Diseases *initiative* (DND*i*), PATH, Medicines for Malaria Venture (MMV), Global Alliance for Tuberculosis Drug Development (TB Alliance), and the Foundation for Innovative New Diagnostics (FIND) tackle the traditionally high cost of R&D and work to bring new NTD, malaria, and tuberculosis products to the market at affordable prices. In contrast to private industry, which funds its R&D through revenues from marketed products as well as investors, PDPs are supported through various mechanisms that include public funding and philanthropy. This funding mechanism allows PDPs to decouple the price of medicines developed from the cost of R&D. Pharmaceutical companies generally employ cost-based pricing to cover the costs of R&D and to generate a profit. Not only do PDPs keep their costs low through efficient collaborations, and smaller clinical trials [98], but once a product is developed, PDPs are not under pressure to generate high-margin profits to cover development costs. Consequently, PDPs can price products in a manner that can be afforded by those in need. In addition to this, by establishing strong partnerships with private industry, PDPs benefit from access to resources such as compound libraries and the pharmaceutical industry’s expertise in getting a product to market. Through this collaborative model, it has been possible to reduce development costs and set prices that can be afforded by low- and middle- income countries (LMICs), thus demonstrating that alternative models for neglected disease R&D are possible [98].

By leveraging world-class infectious disease and biological expertise as well as innovative thinking from various research institutions, and by maximizing the use of the material assets and product development know-how of global pharmaceutical and biotechnology companies, PDPs have made it possible to streamline drug discovery to target diseases of poverty in a coordinated and efficient manner. Not only does this incentivize the sharing of ideas and resources across various sectors as outlined in United Nations Sustainable Development Goal (SDG) 3, but it also successfully addresses SDG 17 by directly engaging the private and public sectors in global partnerships and cooperation. However, due to the limited resources available to support product development for neglected diseases, additional collaborative efforts are needed to fill R&D gaps. Although the Pediatric Praziquantel Consortium is working towards a new pediatric formulation of PZQ, there are currently no PDPs focused on novel schistosomiasis drug discovery, thus necessitating other collaborative models. It is such collaborative agreements and cross-sector-inspired business models that will create a sustainable framework for product development in schistosomiasis and other diseases of poverty. 

## 4. WIPO Re:Search as a Platform for Cross-Sector Collaborations That Accelerate Drug Discovery

New and creative collaborative approaches involving both the for-profit and academic/non-profit sectors are required to catalyze product development for schistosomiasis and other high-priority, low-incentive medical needs. Although many pharmaceutical companies have demonstrated commitment to corporate social responsibility (CSR) through drug donation programs for schistosomiasis and other diseases of poverty, as well as through participation in PDPs and PPPs, additional models that complement those efforts and have a longer-term and more sustainable impact on affected populations are needed. Such models need to not only put in place access plans, so that the resulting products are available to the world’s poorest populations (as is done with PDPs), but also continue to drive innovation and product development through alternative frameworks that build the capacity for researchers in endemic countries to jumpstart their own R&D efforts.

One such collaborative model, WIPO Re:Search, was established in 2011 by the World Intellectual Property Organization (WIPO) and BIO Ventures for Global Health (BVGH), and is supported by eight pharmaceutical companies (Eisai Co., Ltd., GlaxoSmithKline, Johnson & Johnson, Merck KGaA, Merck Sharp & Dohme [MSD; known as Merck & Co., Inc. in the U.S. and Canada], Novartis, Pfizer, and Takeda Pharmaceutical Company, Ltd.). WIPO Re:Search is a global initiative that connects people, resources, and ideas across biotechnology and pharmaceutical companies, governments, and non-profits to accelerate drug, diagnostic, and vaccine development for schistosomiasis, other NTDs, malaria, and tuberculosis [99]. WIPO Re:Search operations are supported by the financial contributions of the eight participating companies. While the companies may have internal R&D programs focused on neglected diseases, they have also made commitments to sharing their intellectual property (IP) assets with WIPO Re:Search investigators all over the world, to accelerate the development of novel products that can one day play a critical role in eliminating diseases of poverty. To date, WIPO Re:Search has 140 members in 40 countries across six continents, and has established 140 collaborations, with 14 of them focused on schistosomiasis.

The WIPO Re:Search consortium is a global PPP platform, which, through BVGH’s targeted partnering approach, catalyzes product development for neglected diseases. BVGH recruits targeted member organizations based on strategic considerations, which may include the need for additional corporate engagement, specialized subject matter expertise, or other resources such as specific screening assays or animal models. Once an academic/non-profit institution joins the Consortium, BVGH reviews its assets, and identifies and connects with researchers who work on neglected diseases. The researchers share their partnering interests with BVGH during introductory calls, enabling the identification of potential collaborators with aligned interests. Once mutual interest in collaborating is established, BVGH introduces the parties to each other and coordinates communications between partners to solidify collaboration details, roles, and responsibilities. Once milestones, timelines, deliverables, and action items are developed and agreed upon, the participating organizations execute material transfer agreements (MTAs) or other legal agreements to begin the collaboration. Once an agreement is in place, BVGH provides partnership support and alliance management to help ensure project success. 

WIPO Re:Search uses IP sharing to incentivize and promote innovative product development and access in areas that have traditionally yielded low profitability, such as neglected diseases. It is through the sharing of IP assets for neglected disease R&D at no cost to the user organization, and by having members agree to the WIPO Re:Search Guiding Principles (detailed below) [100], that the Consortium promotes the accessibility of the resulting products to populations that are disproportionately affected by those diseases. Each legal agreement is executed in accordance with the Guiding Principles, which state that IP is to be shared on a royalty-free basis for R&D of products, technologies, and services to address public health needs for neglected diseases in least developed countries (LDCs), which are low-income countries currently confronting severe structural impediments to sustainable development that are also highly vulnerable to economic and environmental shocks [101]. Further, the Guiding Principles state that, for any products resulting from WIPO Re:Search collaborations, IP owners agree to provide royalty-free licenses for their use and sale in LDCs. IP owners also agree to consider in good faith the issue of product access for all developing countries. Finally, the institution that is using the shared IP is allowed to retain ownership of any new IP that results from WIPO Re:Search collaborations and is encouraged to share those assets with other members of the Consortium.

Through WIPO Re:Search, the most commonly shared IP assets are pharmaceutical company compounds (both targeted libraries and diversity sets) that have the potential to be repurposed as new drugs for schistosomiasis and other neglected diseases. Compounds provided by pharmaceutical companies may have already undergone preclinical or early-stage clinical testing, thereby reducing the number of experiments that need to be conducted to validate the compounds’ safety or metabolic properties. This adds value, as it allows scientists to prioritize compounds with good metabolic and safety data that are more likely to move forward through the drug development pipeline. Alternatively, if a compound has a poor toxicity/safety profile, investigators can save valuable time and resources by focusing their efforts on compounds with better profiles. Further, during the drug development process, pharmaceutical companies synthesize large sets of compounds and structurally similar analogs as inhibitors of human target proteins. Like humans, *Schistosoma* are eukaryotes and thus have similar, yet distinct, cellular targets. Thus, compounds targeting human molecules with weak affinity/activity may have the potential to bind to and inhibit homologous proteins found in parasites such as *Schistosoma*. Such large target-specific compound libraries can be repurposed for schistosomiasis as well as many other diseases. Given that the average time required for a drug to reach the market is 12 years [102], it is important to not only identify ways to reduce the time it takes for that drug to get to market (and potentially the cost of the final product), but also reposition important resources such as compounds, technology platforms, and funding in a way that maximizes the strengths of industry and academic/non-profit scientists.

Since companies are likely to have invested significant time and money in developing compounds that target proteins that are dysregulated in non-communicable diseases such as cancer and hypercholesterolemia, targeting the parasitic homologs of those human proteins can be especially promising for neglected disease drug discovery. The key is to identify homologous target proteins that are essential to the survival of the parasite, and pharmaceutical compounds with biological activity against those homologous targets. One of the earliest schistosomiasis-related collaborations established through WIPO Re:Search began in 2013. Through phenotypic whole-organism screens of various compounds, Dr. Conor Caffrey, then at the University of California, San Francisco (UCSF), found that commercial anti-hypercholesterolemia statin drugs were potent schistosomicidal compounds [103]. The statins had been initially developed to treat patients who suffered from elevated cholesterol levels, but as inhibitors of HMG-CoA reductase (3-hydroxy-3-methyl-glutaryl-CoA reductase or HMGR), they also had the potential to be repurposed to inhibit HMG-CoA reductase activity in other organisms. In particular, *S. mansoni’s* survival and egg-producing abilities had been shown to be dependent on schistosomal HMG-CoA reductase (SmHMGR) activity both in vivo and in vitro [104], and Dr. Caffrey was looking to exploit that vulnerability with inhibitors that specifically targeted this protein. 

In order to screen chemical analogs that could prove to be even more potent against SmHMGR and develop a drug discovery program around this promising validated target, BVGH connected Dr. Caffrey with MSD. As a successful manufacturer of statin drugs, MSD was able to provide a select set of statin analogs to support Dr. Caffrey’s efforts. BVGH also connected Dr. Caffrey with investigators from the Center for Infectious Disease Research (CIDR), and the NIAID-funded Seattle Structural Genomics Center for Infectious Disease (SSGCID) to solve the crystal structure for SmHMGR. In addition, MSD scientists provided scientific input on optimized SmHMGR gene expression methodologies that are being used to express the SmHMGR protein so that the crystal structure can be elucidated. With the crystal structure, Dr. Caffrey, now at the Center for Discovery and Innovation in Parasitic Diseases (CDIPD) at the University of California, San Diego (UCSD), and his collaborators will have the option to optimize the hit compounds utilizing a structure-guided medicinal chemistry approach. 

Based on the model of directing drug discovery efforts by using validated targets essential to parasite survival, BVGH has continued to establish fruitful collaborations that involve the sharing of targeted compounds from WIPO Re:Search company members. Eisai Co., Ltd. has also become involved in schistosomiasis R&D by agreeing to provide an investigator with a calcium channel antagonist, as it has been shown that this type of inhibitor has activity against schistosomula and is capable of significantly reducing the viability of adult worms, alone or in combination with PZQ [105]. Further, in line with the hypothesis that polo-like kinases play an important role in *S. mansoni* gametogenesis [106], Takeda Pharmaceutical Company, Ltd., agreed to support efforts surrounding this target by providing a selective polo-like kinase inhibitor that was originally developed to treat solid tumors. By sharing compounds that have selective activity against a verified target, companies are not only providing chemical analogs that researchers would typically have difficulty accessing, they are also directly accelerating the drug discovery process by avoiding the use of compounds with unknown activities, properties, and targets. The use of natural products is also an area that is quite active for drug discovery. Due to the need for novel drugs against schistosomiasis, screening natural products for schistosomicidal activity could potentially lead to the identification of interesting starting points for further drug development, such as novel chemistry or mechanisms of action. For this reason, BVGH connected a researcher at Swiss TPH with the Griffith Institute for Drug Discovery (GRIDD) to gain access to GRIDD’s Nature Bank [107] and screen natural product extracts for activity against *S. mansoni*.

Although repurposing pharmaceutical compounds is an important strategy for developing novel therapeutics in a cost-effective way, the value of the WIPO Re:Search IP-sharing model in advancing R&D for schistosomiasis and other diseases of poverty goes beyond compounds. The model also involves the sharing of knowledge and expertise. Such sharing is especially impactful when it involves scientists from LMICs, as it not only incentivizes innovation in neglected disease-endemic countries, but it also promotes R&D capacity building. This type of capacity building plays an essential role in changing the global development landscape from donation-based programs to long-term sustainable R&D models in countries that currently lack the necessary resources to successfully jumpstart their own product development efforts. Many LMIC researchers focus their efforts on diseases that affect their countries, but they may not have the product development know-how or infrastructure to advance those efforts to the clinic. WIPO Re:Search provides LMIC scientists with a platform that reduces the access barrier to industry and academic/non-profit IP assets and facilitates the necessary knowledge transfer that will eventually empower and strengthen the scientists’ ability to contribute to product development for diseases that challenge their populations. 

WIPO Re:Search collaborations are enabling knowledge and technology transfer between high-income country organizations and LMIC institutions to bolster schistosomiasis R&D capacity in the latter. In one instance, an investigator from Ghana’s Kwame Nkrumah University of Science and Technology (KNUST) was interested in receiving training in *S. mansoni* cultivation and maintenance and screening of Ghanaian medicinal plants for antiparasitic activity, to enhance KNUST’s research and education programs. BVGH connected the researcher with a faculty member who was at UCSF at the time, and the visiting scientist was able to receive the desired training from a world-renowned schistosomiasis researcher. The collaborative work between these two investigators resulted in a *Journal of Parasitology Research* publication [108]. Other WIPO Re:Search collaborations involve the sharing of reagents critical to LMIC investigators’ work plans. For example, Dr. Floriano Paes Silva Junior, an investigator at FIOCRUZ (Brazil), was interested in validating a group of genes as essential to *S. mansoni* through RNA interference (RNAi) and determining whether those proteins could be potential drug discovery targets. BVGH connected the researcher to Alnylam, a pharmaceutical company focused on RNAi therapeutics. Alnylam agreed to design and synthesize optimized siRNAs that would enable the FIOCRUZ team to knock down the genes and determine whether their loss affects parasite viability. An additional example involved the sharing of a recombinant schistosome protein that Dr. Moustapha Mbow at Cheikh Anta Diop University (Senegal) was interested in using to assess in vitro immune responses to the protein. BVGH connected Dr. Mbow to Dr. Michael Hsieh at the Biomedical Research Institute (BRI), who expressed, purified, and shared the protein. This collaboration has led to a joint grant submission.

## 5. Conclusions

To boost the number of quality novel candidates that enter clinical-stage development, the schistosomiasis product development pipeline needs to be continuously fueled by early-stage research. It is during this stage of the product development process that WIPO Re:Search can have the greatest impact by establishing collaborations between for-profit and academic/non-profit institutions. The pharmaceutical industry not only engages in global health initiatives through its drug donation programs, but it also plays an important role in driving R&D for diseases of poverty. However, many academic/non-profit researchers remain unaware of the partnership opportunities that are available to them to drive their own research forward. The WIPO Re:Search consortium can bridge the gap between academic/non-profit and industry scientists to reduce duplicative efforts and encourage synergy to develop vaccines, diagnostics, and drugs for schistosomiasis and other neglected diseases. Furthermore, through the capacity building activities of WIPO Re:Search, LMIC scientists are not only able to gain valuable expertise, but also to use that expertise to develop new skills, accelerate their research, and advance their careers. The expertise gained through such collaborations provides scientists with a distinct advantage that can result in additional funding to reach important milestones. It is through the mutually beneficial model of WIPO Re:Search, where product development risk and cost are distributed among various key stakeholders, that a tangible and significant impact can be made to eliminate schistosomiasis and other diseases of poverty. 

As the fight against schistosomiasis continues, the involvement of private industry and their critical contributions of assets and expertise should be causes for optimism. While promising industry collaborations continue to advance towards novel products and tools that will play a key role in the elimination of schistosomiasis, it is important to remember that this task is too great to be overcome by a single sector. As pharmaceutical companies continue to invest resources and funding to combat schistosomiasis, it is important for these efforts to receive the support of various stakeholders such as governments and international funding agencies. Through a concerted effort that distributes risk, and leverages the strengths and resources of the private and public sectors, every day brings us closer to winning the fight against schistosomiasis.

## Figures and Tables

**Table 1:** Vaccines in development (Phase I-III clinical trials).

Product	Antigen	Phase of Clinical Development ^1^	Targeted Species
Bilhvax^®^	*Sh*-GST28	Phase III	*S. haematobium*
*Sm*-14	*Sm*-14	Phase II	*S. haematobium* *S. mansoni*
Alhydrogel^®^	*Sm*-TSP-2	Phase Ib	*S. mansoni*
*Sm*-97 paramyosin	*Sm*-97	Phase I	*S. mansoni*
Calpain^®^	*Sm*-p80	Phase I	*S. mansoni*

^1^ Vaccines in preclinical stages of development are not included.

**Table 2:** Applications of diagnostic platforms.

Diagnostic Platform	Qualities	Mechanism of Detection	Stage of Elimination Program for Application
Microscopic Detection	Recommended by WHO	Microscopic egg counting from fecal or urine sample	Mapping prevalence
	Low sensitivity: egg shedding is often not observed upon infection or in instances of low infection		
Lateral flow test	Field deployable	Antigen detection: indication of active infection	Mapping prevalence
			Monitoring the impact of MDA
	Minimal training requirements		
			Informing reduction/stopping of MDA
	Relatively low cost		
	Incorporating a reader may improve test performance metrics		
		Antibody detection: indication of past or present exposure to disease	Post-elimination surveillance ^1^
Molecular Diagnostic Testing	Implementation is limited to a laboratory setting	Nucleic acid detection: indication of active infection	Informing reduction/stopping of MDA
	Requires sample preparation		
	Highly sensitive and specific		
	Too expensive for mass distribution		
	Ability for multiplex pathogen detection		
	Rapid turnaround time		

^1^ Antibody-based technology is limited due to its inability to differentiate between ongoing and previous infections, as the detected antibodies remain present even after clearance of parasites. Antibody-based diagnostics have application for post-elimination surveillance by monitoring antibody prevalence in age cohorts that were born after elimination was certified; if antibodies are detected in this youth population, then there has been a re-emergence of the disease [70] (PATH, Diagnostics for Neglected Tropical Diseases).

**Table 3:** Therapeutics in development.

Product	Phase of Development	Targeted Species
Co-Arinate FDC^®^	Phase III	*S. haematobium*
Pediatric PZQ	Preclinical	*S. haematobium* *S. mansoni* *S. japonicum*
Miltefosine (MFS)	Preclinical	*S. mansoni*
